# The mediating role of ego depletion in the relationship between state anxiety and academic procrastination among University students

**DOI:** 10.1038/s41598-024-66293-6

**Published:** 2024-07-06

**Authors:** Junqiang Fan, Yongjie Cheng, Ming Tang, Yuxin Huang, Jingjing Yu

**Affiliations:** 1 School of Economics and Management, Zhejiang University of Science and Technology, Hangzhou, 310023 Zhejiang China; 2 School of Environment and Natural Resources, Zhejiang University of Science and Technology, Hangzhou, 310023 Zhejiang China; 3 Anji Campus of Zhejiang, University of Science and Technology, Anji, 313301 Zhejiang China; 4 Hangzhou Xixi Hospital, Hangzhou, 310023 Zhejiang China

**Keywords:** State anxiety, Ego depletion, Academic procrastination, Gender, University students, Health services, Occupational health, Human behaviour

## Abstract

The issue of academic procrastination is highly prevalent among university students. It not only has a deterimental effect on students' academic performance but also poses a risk to their physical and mental well-being. Anxiety, as a negative emotion, has attracted researchers’ attention in relation to academic procrastination. Research indicates a correlation between state anxiety and academic procrastination, but the underlying mechanisms that drive this association remain unclear. When individuals experience ego-depletion, it can lead to psychological exhaustion, subsequently leading to procrastination. Gender role conceptions, shaped by sociocultural and psychological mechanisms, have profound implications on individuals’ cognition, emotions, and behaviors. This study primarily aims to explore the relationship between state anxiety and academic procrastination among university students, with a particularly focus on the mediating role of ego-depletion and the moderating role of gender. A survey using the State Anxiety Scale, Ego-Depletion Scale, and Irrational Procrastination Scale was administered to 3370 undergraduates. State anxiety shows positive correlations with ego depletion and academic procrastination (*r* = 0.665, *p* < 0.01; *r* = 0.491, *p* < 0.01), while ego depletion is also positively linked to academic procrastination (*r* = 0.500, *p* < 0.01). State anxiety serves as a positive predictor of academic procrastination, with a confidence interval of 95% [0.626, 0.696]; additionally, ego depletion partially mediates the relationship between state anxiety and academic procrastination, with a confidence interval of 95% [0.168, 0.251]. Gender acts as a moderator in directly predicting the impact of state anxiety on academic procrastination and in the latter stage of mediating the effect of ego depletion. State anxiety can significantly and positively predict academic procrastination among university students. Ego-depletion partially mediates the relationship between state anxiety and academic procrastination. The direct predictive effect of state anxiety on academic procrastination, as well as the mediating role of ego-depletion, is moderated by gender. This provides educators and university students themselves with reference for addressing the issue of academic procrastination.

## Introduction

Procrastination refers to the voluntary delay in initiating or completing a specific task or plan^1^. As society advances rapidly, the issue of procrastination becomes increasingly serious and prominent. Academic procrastination, as an extension of procrastination within the learning context, involves the purposeful delay of tasks that must be completed in the academic setting. The identification of academic procrastination typically involves meeting three criteria: delay, non-necessity, and the production of adverse consequences^2^. Academic procrastination is a primary manifestation of academic challenges among university students. Extensive investigations conducted by scholars from diverse national and international backgrounds reveal the pervasive existence of academic procrastination within the university student population. Over half of high school and university students exhibit tendencies toward academic procrastination^1,3^. Chinese studies indicate that, concerning pre-exam review and self-directed study, university students exhibit procrastination rates of 47.1% and 48.1%, respectively^4^. Furthermore, a striking 96.6% of university students, as reported by Gao^5^, exhibit tendencies toward moderate to high levels of academic procrastination. Solomon and Rothblum postulated that procrastination embodies an intricate psychological and behavioral pattern emerging through the interaction of cognitive, emotional, and behavioral elements. Presently, contemporary scholars have undertaken comprehensive exploration into the determinants of academic procrastination, employing diverse perspectives such as trait theory, motivational theory, and self-regulation theory^2,6,7^. Broadly speaking, academic procrastination is commonly perceived as possessing unfavorable implications and is characterized as an irrational behavior^8^. This phenomenon has been correlated with detrimental impacts on students’ academic performance^9^, as well as their physical and mental well-being, and emotional experiences^10^. Consequently, an in-depth exploration of the influencing factors and mechanisms underlying academic procrastination among university students not only enhances pertinent theoretical frameworks but also provides guidance for implementing interventions aimed at alleviating academic procrastination.

### The relationship between state anxiety and academic procrastination

Anxiety denotes an unpleasant emotional state^11^. Spielberger et al.^12^ categorizes anxiety into trait anxiety and state anxiety based on its stability and persistence. The former refers to an individual anxiety tendency as a personality trait with individual differences, while the latter denotes anxiety triggered by a specific situation, presenting as a temporary and immediate state with a certain intensity level^13^. Individuals exhibiting elevated trait anxiety levels demonstrate an increased propensity for perceiving threatening stimuli, thereby triggering elevated levels of state anxiety^14^. Current research often links anxiety to personality, cognitive styles, parent–child relationships, attention, coping mechanisms, and predominantly explores the negative impacts of anxiety^15,16^. Procrastination behavior is commonly linked with negative emotions, and the relationship between anxiety and procrastination is increasingly gaining researchers’ attention. Rothblum et al.^17^ propose that procrastination results from avoiding unpleasant emotions such as anxiety. A wealth of empirical studies suggest that anxiety is a crucial predictor of procrastination^18,19^. Aligned with the theory of motivation and engagement, anxiety is a significant influencing factor on learning engagement. Certain individuals frequently engage in procrastination or abstain from studying owing to adverse perceptions of anxiety and other unfavorable emotions, resulting in diminished learning engagement^20,21^. Academic procrastination is commonly associated with negative emotions, particularly anxiety. When students face tasks of certain difficulty, they may experience state anxiety and subsequently divert their attention to other enjoyable activities to alleviate this anxiety, resulting in the postponement or delay of academic tasks. Thus, the higher the level of state anxiety correspond the greater degree of academic procrastination. Zhan^22^ found that academic procrastination is closely related to state anxiety, and state anxiety partially mediates the link between irrational beliefs and academic procrastination. In summary, based on prior research, Individuals prone to initiating and experiencing anxiety during tasks tend to employ procrastination as a defense mechanism to alleviate discomfort. However, this strategy fails to truly eliminate the imbalance in the individual state, exacerbating negative emotions and leading to more severe procrastination behaviors, creating a vicious cycle^23^. Thus, the study formulates hypothesis 1: state anxiety is positively related to diabetes debt.

### Ego depletion as a mediating role

The theory of ego depletion posits that individuals undergo a depletion of self-control capacities following engagements in activities demanding self-control resources. This depletion manifests ego depletion^24^. Individuals make their words and deeds conform to social norms through self-control. Factors including subjective cognition, emotions, personality traits, intimate relationships will cause self-depletion^25^. In our study, on one hand, anxiety may lead to ego depletion. Being a negative emotion, anxiety can contribute to increased levels of ego depletion^26,27^, resulting in a decline in self-control capacity. In addition, individuals experiencing high state anxiety are inclined to focus on their internal feelings, thereby elevating psychological load and depleting mental resources^28^. Besides, ego depletion constitutes a critical risk factor for individual social adaptation and mental health^29^. When an individual is experiencing ego depletion, it will cause psychological exhaustion, and the individual tends to produce unconscious or automatic behaviors, such as luxury consumption, alcohol dependence, and addiction to the Internet, which is regarded as a contributing factor to academic procrastination^30,31^. Some scholars have elucidated the internal mechanism through which ego depletion contributes to academic procrastination. When self-control resources are excessively utilized, resulting in ego depletion, the deliberative system becomes inhibited. Consequently, individuals tend to rely more on intuition when making decisions, exhibiting a propensity for irrational risk-taking behaviors. In the context of academic procrastination, individuals experiencing high levels of ego depletion are inclined to irrationally assess the negative consequences of procrastination. They make decisions based on habitual patterns, derive immediate benefits from procrastination, thereby exacerbating academic procrastination behaviors^32^. Based on the above analysis and the theory of ego depletion, this study proposes hypothesis 2: Ego depletion has the mediating function in the relationship between state anxiety and academic procrastination.

### Gender as the moderating factor

Gender role concepts formed based on social culture and psychological mechanisms play an important guiding role in individual cognition, emotion and behavior. From a physiological standpoint, variances in brain structure and function across genders may contribute to cognitive distinctions, and these divergences in cognitive processing, in turn, are likely to influence behavioral disparities^33^. An electroencephalogram investigation conducted by Proverbio et al.^34^ demonstrated that, compared to men, women displayed larger N2 amplitudes in response to negative stimuli sourced from the International Affective Picture System, particularly stimuli associated with humans. This observation suggests that women manifest stronger emotional reactions to negative stimuli. There are significant gender differences in occupational anxiety among young university teachers, with female teachers exhibiting significantly higher levels of anxiety than male teachers^35^. Moreover, relevant research indicates significant gender variations in self-control, specifically noting that females exhibit markedly higher self-control than males^36^. As individuals age, women tend to demonstrate superior self-regulation abilities in comparison to men^37^. Regarding gender differences in academic procrastination, current research has not reached a unified conclusion and often treats gender as a control variable. For example, the findings of research about junior high school students by Zhu et al. also indicated the absence of statistically significant gender difference in academic procrastination, and the occurrence of procrastination behaviors during the learning process was similar for both males and females^38^. In contrast, a study by Li and Yang concluded that there are significant gender differences in academic procrastination and self-control among junior high school students, with male exhibiting much more serious academic procrastination than female^39^. When faced with academic tasks, males and females may have different mechanisms for producing academic procrastination due to differences in cognitive processing. In light of this, this study will exploratorily analyze the moderating role of gender, proposing hypothesis 3: The direct predictive function of state anxiety on academic procrastination and the mediating impact of ego depletion on the first half path and the second half path are both moderated by gender.

### The current study

**Figure 1:**
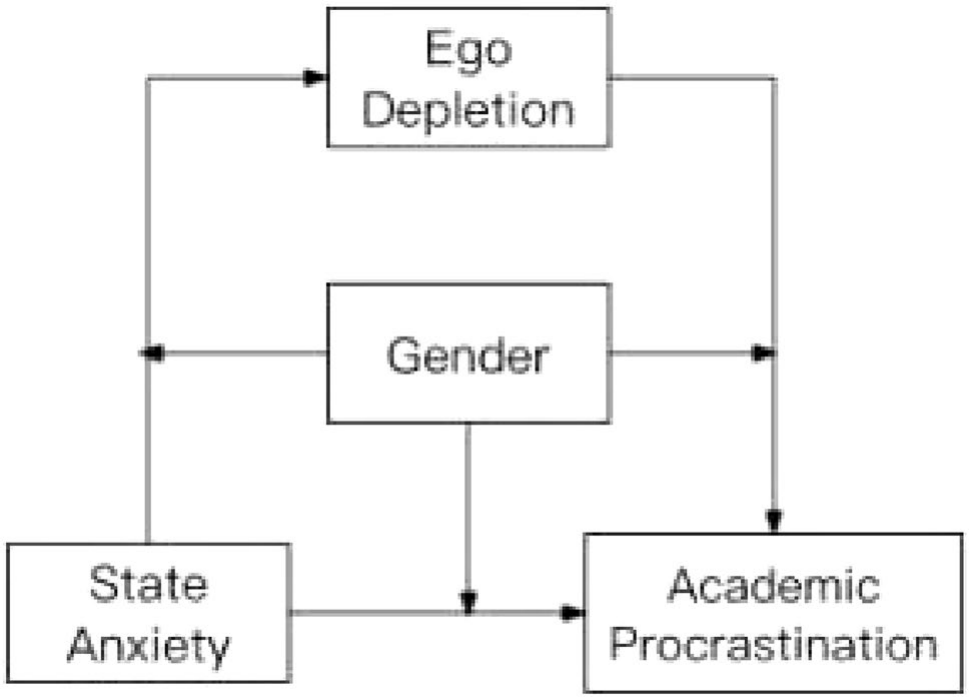
A moderated mediation model.

In this study, based on the preceding discussion, a moderated mediation model is developed (Fig. 1) to explain how state anxiety influences university students’ academic procrastination and the potential mechanisms of ego depletion and gender. Undoubtedly, existing academic achievements both domestically and internationally on academic procrastination have provided a theoretical foundation for this study. However, there is a lack of systematic research on the mediating role of ego depletion in the relationship between state anxiety and academic procrastination. The mechanism of how state anxiety affects academic procrastination through mechanisms such as ego depletion has not been thoroughly explored^22^, and the moderating role of gender in this relationship has not been sufficiently studied^40^. Addressing these gaps will enhance our understanding of the factors contributing to academic procrastination and provide information for targeted intervention measures. Based on this, three hypotheses are proposed. Hypothesis 1: There is a positive correlation between state anxiety and academic procrastination. Hypothesis 2: Ego depletion plays a mediating role between state anxiety and academic procrastination. Hypothesis 3: The direct predictive effect of state anxiety on academic procrastination and the anterior and posterior paths of the mediating role of ego depletion are moderated by gender. It aims to provide a new theoretical perspective on the potential factors of academic procrastination of university students and effectively intervene in its negative impact.

## Methods

### Participants

The inclusion criteria were current undergraduate university students, all of whom provided informed consent and voluntarily participated in the research with strict protection participants’ privacy. Exclusion criteria were students with major physical illnesses or significant psychological trauma, such as the recent serious illness or death of a family member. According to the Kendall’s sample estimation method, the sample size should be 5–10 times the number of independent variables. This study included 8 general information items, 9 items on the Academic Procrastination Scale, 20 items on the State Anxiety Scale, and 5 items on the Ego Depletion scale, resulting in a total of 42 variables. Based on the tenfold rule, the estimated sample size was 420 cases. Accounting for a 20% rate of invalid questionnaires, the final sample size was determined to be no less than 525 cases. To obtain more data, a larger sample size was selected for the questionnaire survey, ultimately including 3832 participants, providing sufficient power to detect the hypothesized effects. After launching online survey through the platform called Questionnaire Star, this study has collected 3,832 pieces of data. After excluding abnormal data with a repetition rate of more than 70% of answer options and answer times beyond three standard deviations, 3,370 valid questionnaires were obtained, with an effective rate of 87.9%. 2216 were males (65.76%) and 1154 were females (34.24%); 1468 (43.56%) came from city, 622 (18.46%) came from towns, and 1280 (37.98%) came from rural areas. The mean age of participants was 19.41 (SD = 4.48).

### Measure

#### Academic procrastination scale

The irrational procrastination scale compiled by Steel^1^ and modified by Ni et al.^6^ was employed to analyze of irrational procrastination among university students in completing academic tasks, with questions such as “I failed to complete the task within the specified time limit.” Nine items in this 5-point scale include statements such as “I failed to complete the task within the specified time limit.”, ranging from “almost never” (1 point) to “always” (5 points). A higher overall score indicates greater irrational procrastination. The Cronbach’s α was 0.725.

#### State anxiety scale

This study used the subscale of the State-Trait Anxiety Inventory compiled by Spielberger et al.^12^ and revised by Zheng and Li^41^: State Anxiety Inventory (S-AI), with questions such as “I feel calm”. The instruction is “Please choose the most appropriate feeling for you at this moment”. This 4-point subscale has a total of 20 items ranging from “not at all” (1 point) to “very obviously” (4 points). Higher score shows the greater level of state anxiety. The Cronbach’s α of the state anxiety subscale was 0.939.

#### Ego depletion scale

This study employed the ego depletion scale developed by Lin and Johnson^42^. This scale has 5 items in total, including “I feel exhausted”, “I can’t concentrate right now”, “It takes a lot of effort for me to focus on certain things now”, “I am deficient in energy”, “I feel like I have no willpower”, from 1 point (completely inconsistent) to 5 points (completely consistent), which is scored in a positive direction. Higher scores indicate higher ego depletion. The Cronbach’s α of the ego depletion scale was 0.927.

### Procedure

This study employed an online questionnaire to conduct a comprehensive survey. Participants were instructed to identify various scenarios involving irrational procrastination, encompassing tasks such as writing term papers, completing coursework, managing academic responsibilities, and handling assigned tasks from departments or classes, as well as self-assigned learning tasks. All questions were mandatory, ensuring the absence of missing data.

### Data analysis

Data processing utilized SPSS 20.0 statistical software. The validity of each scale underwent confirmatory factor analysis (CFA). The mediation model was analyzed using the SPSS macro PROCESS v3.5 with the Bootstrap method for bias correction.

### Ethics and consent declaration

The study was conducted according to the guidelines of the Declaration of Helsinki and approved by the Research Ethics Committee of Zhejiang University of Science and Technology. The participants signed the informed consent to participate in this study.

## Results

### Common method bias test

Since the data in this study were all derived from self-reports, which could potentially lead to common method bias, the Harman’s single-factor test was adopted to assess this issue. The results showed that there were a total of 8 factors with eigenvalues greater than 1, and the first factor explained 33.56% of the variance, which is less than the critical threshold of 40%^43^. Therefore, there is no significant common method bias in this study.

### Descriptive and correlational analysis

Tables 1 and 2 display the mean, standard deviation, and correlation matrix for each variable. Significant positive correlations were observed among state anxiety, ego depletion, and academic procrastination. This indicates a close relationship between the study variables, warranting further analysis. Additionally, a remarkable positive correlation was identified between gender and state anxiety (*p* < 0.05), as well as ego depletion (*p* < 0.01). Given the relevance of place of residence to key study variables, it is included as a control variable.

**Table 1:** Descriptive statistics.

Variable	Minimum Value	Maximum Value	M	SD	Skewness	Kurtosis
1 Gender	1	2	1.34	0.475	0.665	− 1.560
2 Place of residence	1	3	1.94	0.902	0.110	− 1.762
3 Age	16	28	19.32	1.060	1.850	7.680
4 State anxiety	20	80	40.57	10.822	− 0.039	− 0.195
5 Ego depletion	5	25	12.81	5.048	0.093	− 0.795
6 Academic procrastination	9	40	23.18	5.333	− 0.209	0.006

**Table 2:** Correlation analysis.

Variable	1	2	3	4	5	6
1 Gender	1					
2 Place of residence	0.057*	1				
3 Age	0.103**	0.124**	1			
4 State anxiety	0.054*	0.049*	0.049*	1		
5 Ego depletion	0.089**	0.068**	0.066**	0.665**	1	
6 Academic procrastination	0.014	0.014	0.044	0.491**	0.500**	1

*M* mean, *SD* standard deviation.

*p < 0.05, **p < 0.01, ***p < 0.001 (similar for all following).

### Mediation model test

With gender and place of residence as controlled factors, this study used Model 4 in the SPSS macro PROCESS^44^ to test the mediating effect of ego depletion between state anxiety and academic procrastination. Table 3 shows the result that state anxiety can obviously and positively predict ego depletion (*β* = 0.660, *t* = 36.290, *p* < 0.001) and academic procrastination (*β* = 0.492, *t* = 23.077, *p* < 0.001). When the mediating variable is included, the direct predictive effect of state anxiety on academic procrastination is still significant (*β* = 0.283, *t* = 10.314, *p* < 0.001), and the positive predictive effect of ego depletion on academic procrastination is also obvious (*β* = 0.317, *t* = 11.511, *p* < 0.001).

**Table 3:** Mediation model testing.

Predictors	Ego depletion		Academic procrastination		Academic procrastination	
	*β*	*t*	*β*	*t*	*β*	*t*
Gender	0.052	2.859**	− 0.012	− 0.566	− 0.029	− 1.387
Place of residence	0.033	1.819	− 0.009	− 0.423	− 0.020	− 0.949
State anxiety	0.660	36.290***	0.492	23.077***	0.283	10.314***
Ego depletion					0.317	11.511***
*R*	0.668		0.491		0.544	
*R*^*2*^	0.446		0.241		0.296	
*F*	451.105		177.784		176.893	

*Note* Each variable is standardized into the regression equation, the same below.

Furthermore, the Bootstrap 95% confidence intervals of the direct effect of state anxiety on academic procrastination and the mediating effect of ego depletion do not encompass zero (Table 4). This signifies that state anxiety can not only directly predict academic procrastination, but also through the mediator of ego depletion role in predicting academic procrastination behavior. The direct effect (0.283) and the mediation effect (0.209) respectively account for 57.49% and 42.51% of the total effect (0.492).

**Table 4:** Mediation effect testing results.

	Effect value	Boot SE	Boot CI lower	Boot CI upper	Relative effect
Total effect	0.492	0.023	0.448	0.536	–
Direct effect	0.283	0.018	0.626	0.696	57.49%
Mediation effect	0.209	0.021	0.168	0.251	42.51%

### Test of the moderating effect of gender

To delve deeper into gender distinctions in academic procrastination, this study employed Model 15 in the PROCESS plug-in to scrutinize the moderating role of gender. According to the moderated mediation model testing method recommended by^45^, all variables are initially standardized, and gender is coded as a dummy variable (1 for males and 2 for females) to assess the moderating effect of gender. The results are presented in Table 5. Upon integrating gender into the model, the interaction term between state anxiety and gender did not exhibit a significant predictive effect on ego depletion (*β* = 0.013, *t* = 0.712, *p* > 0.05). However, both the interaction term between state anxiety and gender and the interaction term between ego depletion and gender demonstrated significance in predicting academic procrastination (*β* = − 0.096, *t* = − 3.466, *p* < 0.001; *β* = 0.099, *t* = 3.493, *p* < 0.001) .

**Table 5:** Moderated mediation model testing.

Regression equation		Overall Fit Index			Regression coefficient and significance	
Outcome variable	Predictor variable	*R*	*R*^*2*^	*F*	*β*	*t*
Ego depletion		0.668	0.446	338.356		
	Gender				0.052	2.833**
	Place of residence				0.033	1.821
	State anxiety				0.660	36.248***
	State Anxiety × Gender				0.013	0.712
Academic procrastination		0.550	0.302	121.197		
	Gender				− 0.031	− 1.528
	Place of residence				− 0.020	− 0.969
	State anxiety				0.277	10.112***
	Ego depletion				0.325	11.820***
	State anxiety × gender				− 0.096	− 3.466***
	Ego depletion × gender				0.099	3.493***

**Figure 2:**
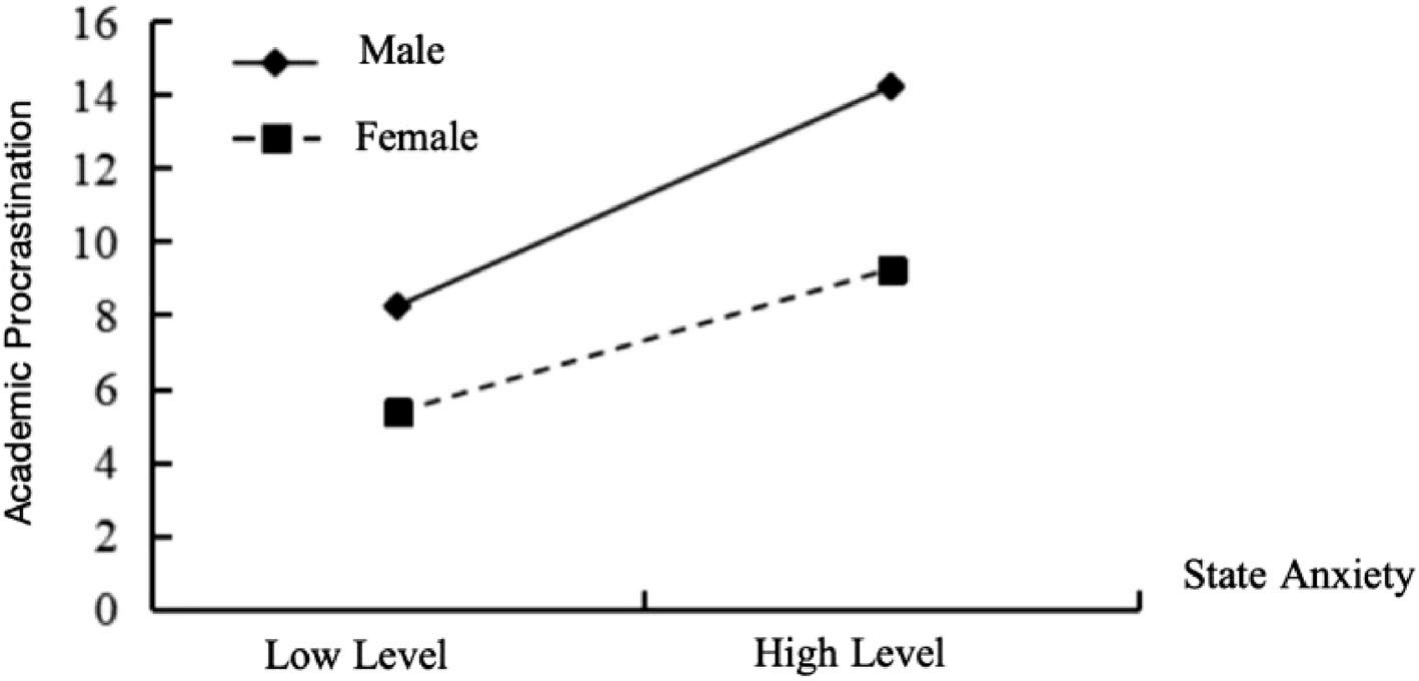
The moderating effect of gender on state anxiety on university students’ academic procrastination.

**Figure 3:**
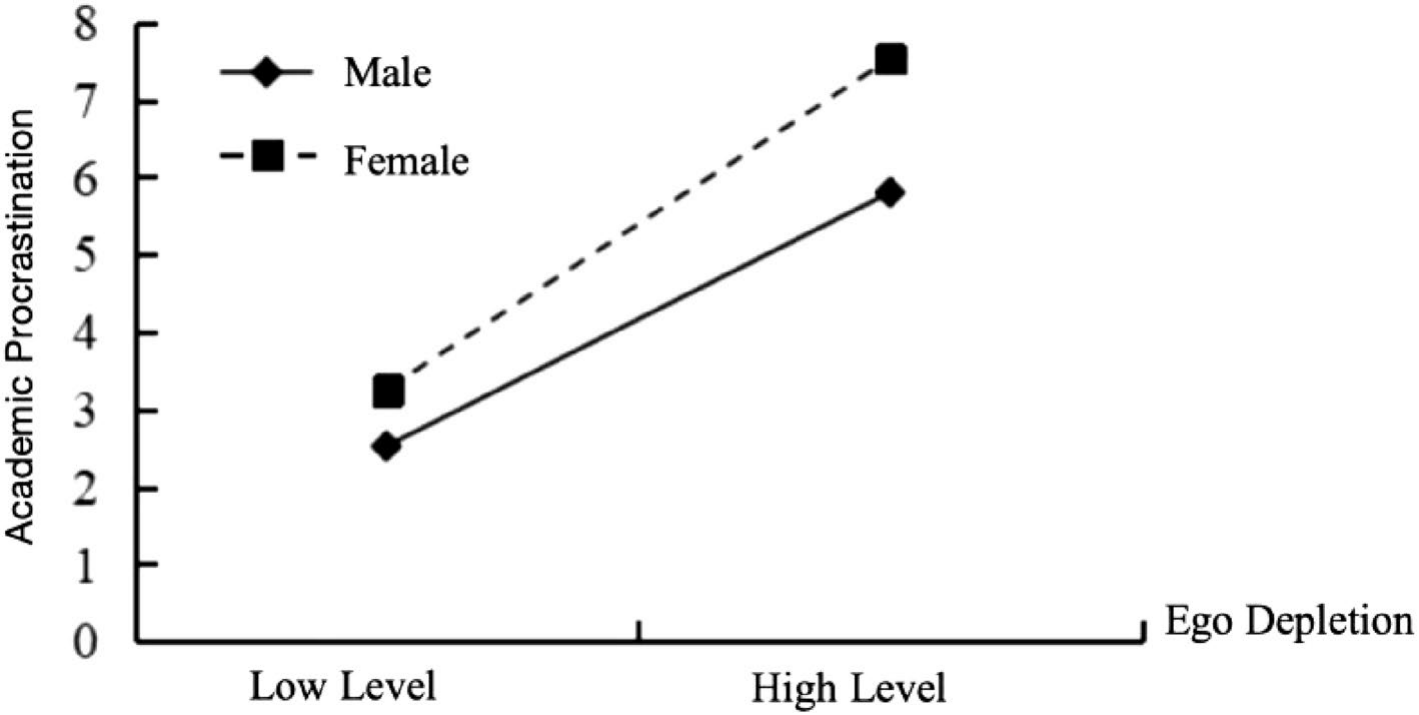
The moderating effect of gender on ego depletion on university students’ academic procrastination.

As illustrated in Fig. 2, when compared with females (*B*_*simple*_ = 0.144, *t* = 2.993, *p* < 0.01), the positive predictive effect of state anxiety on academic procrastination is more pronounced in males (*B*_*simple*_ = 0.346, *t* = 10.398, *p* < 0.001). Additionally, Fig. 3 demonstrates that gender also moderate the influence of ego depletion on academic procrastination. In contrast to males (*B*_*simple*_ = 0.254, *t* = 7.724, *p* < 0.001), females exhibit a more prominent positive prediction of academic procrastination through their level of ego depletion. (*B*_*simple*_ = 0.461, *t* = 9.312, *p* < 0.001).

Taken together, state anxiety significantly mediates the mediating effect of ego depletion on university students’ academic procrastination. The determination index (INDEX) is 0.138 (SE = 0.042), 95% CI = [0.056, 0.221].

Table 6 indicates that among male participants, the positive mediating effect of ego depletion is statistically significant (ab = 0.168, SE = 0.026, 95% CI [0.119, 0.220]); for female, the positive mediating effect of ego depletion is also significant. In comparison with male participants, the mediating effect of ego depletion is notably stronger among females (CONTRAST = 0.138, SE = 0.042, 95% CI [0.056, 0.221]).

**Table 6:** Mediating role of ego depletion in subjects of different genders.

Mediating Variable	Gender	Effect Value	Boot SE	Boot CI Lower	Boot CI Upper
Ego depletion	Male	0.168	0.026	0.119	0.220
	Female	0.306	0.035	0.238	0.375
	Group difference	0.138	0.042	0.056	0.221

## Discussion

The primary objective of this study is to investigate the relationship between state anxiety and academic procrastination among university students, with particular emphasis to the mediating role of ego depletion and the moderating role of gender. Based on the current research landscape of academic procrastination among university students and their academic lives, a moderated mediation model was developed from the perspective of limited self-control theory to explore the impact of state anxiety on academic procrastination behavior and its internal mechanism, which is beneficial to better understand the contributing factors of academic procrastination among university students, and provides empirical evidence for intervention in academic procrastination.

### The relationship between state anxiety and academic procrastination

A positive predictive relationship between state anxiety and academic procrastination among university students has been observed. It indicates that higher levels of state anxiety in university students are associated with a greater likelihood of engaging in academic procrastination behaviors, which supports hypothesis 1. It also aligns with previous findings^19,46,47^, suggesting that state anxiety is a crucial predictive factor for the occurrence of academic procrastination. In accordance with the Anxiety Activation Model^48^, it posits that individuals, when experiencing unpleasant emotions, engage in certain behaviors to alleviate the adverse experiences associated with these emotions. The emotional aspect of state anxiety may be alleviated through academic procrastination, implying a potential influence of state anxiety on academic procrastination. Moreover, according to the Emotional Regulation Theory of procrastination^49^, when individuals are confronted with a highly challenging task and the anxiety elicited by the task becomes overwhelming, procrastination can be viewed as a self-protective mechanism to defend against the discomfort caused by excessively high anxiety levels. Simultaneously, the Transactional Model of Stress and Coping also proves anxiety is a crucial predictive factor for triggering procrastination behaviors. Therefore, state anxiety directly causes the occurrence of academic procrastination in university students. Based on this, from the perspective of educators, alleviating various forms of potential anxiety emotions among students, such as academic pressure, graduation pressure, and employment pressure, can effectively reduce the likelihood of academic procrastination. From the students’ perspective, they should be aware of the correlation between state anxiety and academic procrastination. Students should actively regulate their emotions, reduce unnecessary anxiety, and avoid academic procrastination behaviors, thereby preventing a vicious cycle of academic procrastination adversely affecting their mental health.

### The mediating role of ego depletion

This study has identified that state anxiety predicts the academic procrastination behavior of university students through the mediating effect of ego depletion. When individuals encounter negative emotions, they may deplete restricted self-control resources to regulate these negative feelings^50–52^. This implies that the negative emotions and cognitions triggered by anxiety will consume the individual’s limited psychological energy or self-control resources, which results in ego depletion^53^. In turn, insufficient self-control resources contribute to procrastination and other deviant behaviors. This study explores the mediating mechanism of state anxiety influencing university students’ academic procrastination from a new perspective of ego-depletion theory. While anxiety itself can lead to deviant behaviors, the regulation of negative emotions and stress coping can also deplete psychological resources, heightening the risk of deviant behaviors^25^. Yu et al.^54^ suggest that ego depletion plays a mediating role between unethical tasks and work procrastination, which provides enlightenment for preventing or reducing procrastination behaviors. By investigating the mediating role of ego depletion, this study offers an integrated perspective for understanding the mechanism through which state anxiety affects academic procrastination. This approach incorporates previously examined emotional variables into the self-depletion framework, enriching our understanding of the complex interplay between anxiety, ego depletion, and academic procrastination. Therefore, from an educator’s perspective, practical intervention strategies can be implemented, such as the provision of training programs focused on stress management techniques and the execution of self-control enhancement plans. For individual students, self-emotion regulation is crucial to reduce ego depletion. This can be achieved through participating in diverse activities to foster positive emotions, actively empowering oneself by maintaining a positive mindset, and strengthening interpersonal relationships to enhance external support. Collectively, these approaches are likely to contribute to reducing academic procrastination among university students.

### The moderating effect of gender

This study further examined whether gender moderates the pathways of “state anxiety → ego depletion → academic procrastination behavior of university students” in the first half, the second half, and the direct path. Hypothesis 3 was partially verified. Initially, gender has the moderating function between state anxiety and university students’ academic procrastination. Male students are more susceptible to the impact of state anxiety on academic procrastination. This might be the result of the higher perception of stress in male students, compared to their female counterparts, as elevated stress perception can trigger anxiety in individuals^55^. Moreover, females exhibits more diversified coping mechanisms for anxiety, being more inclined to alleviate anxiety through expression, while males experiencing anxiety tended to self-isolate and withdraw^56^. It also unveils that gender moderates the impact of ego depletion on university students’ academic procrastination behavior. In comparison to males, females are more susceptible to the influence of ego depletion. According to the response style theory, females tend to engage in reflective responses, encoding negative information more deeply and thoroughly, making it challenging to disengage attention from negative emotions^57^. Females are also more prone to the influence of social evaluations^58^, adhering to societal expectations and role-playing, which consumes a portion of self-control resources. Individuals in an anxious state impair the function of the prefrontal lobe, thereby limiting advanced cognitive processing functions. The brain subsequently shifts to a bottom-up response, mediated by the amygdala and subcortical structures. This regulatory mechanism aids individuals in coping with dangerous situations by heightening alertness to potential threats. However, activities requiring deep cognitive processing demand greater cognitive effort^59^. Consequently, heightened anxiety levels cause increased self-depletion in individuals. As such, gender does not significantly moderate state anxiety and ego depletion in the first half of the mediation model proposed in this study. Therefore, particular attention should be directed towards males experiencing elevated levels of state anxiety and females exhibiting increased self-depletion, as they are more prone to academic procrastination.

## Significance and limitations

### Significance

The moderated mediation model not only elucidates the cognitive mechanism through which state anxiety leads to university students’ academic procrastination behavior, but also reveals gender differences in this cognitive mechanism. This study possesses both academic and practical value. It aids educators in understanding the causes of academic procrastination among university students and provides them with timely and effective academic and psychological guidance. Additionally, it also benefits college student groups in better coping with anxiety and adapting to demanding academic life. It offers individuals insight into their procrastination behavior and provides assistance in reducing such behaviors. In terms of academic value, this model addresses both how state anxiety influences academic procrastination behavior and the conditions under which the predictive function of state anxiety on academic procrastination and ego depletion as a mediator are more pronounced. From the perspective of practical application, it contributes to the formulation of effective intervention strategies for university students’ academic procrastination behavior. Firstly, effective identification and focused attention to students with high state anxiety should be emphasized. Conducting psychological health education courses can help students develop a proper understanding of anxiety. Secondly, it is necessary to create a diverse campus environment for students and cultivate their interests in their life, so as to decrease the negative impact of ego depletion. Lastly, the institution should systematically assist students in career planning to help them delineate their positioning, unique capabilities, and learning objectives in a phased manner.

### Limitations

This study has the following limitations: (1) The utilization of a cross-sectional survey design in this research has limitations in making causal inferences.. Although we speculated on the impact of state anxiety on academic procrastination, there are still other potential variables that may have an impact on the research results. To more accurately know the effect of state anxiety on academic procrastination, future research could adopt specific longitudinal or experimental designs to further investigate the degree of academic procrastination among university students under different levels of anxiety, and determine the mechanism of state anxiety’s influence on academic procrastination. (2) This study solely examines gender moderates the relationship between state anxiety and academic procrastination, neglecting other potential individual difference factors such as regional disparities and family economic status. Subsequent research could further discuss moderating role of other individual difference factors on the relationship between state anxiety and academic procrastination, in order to comprehensively understand the mechanism of their interaction.. (3) The sample in this study is confined to university students, limiting the generalization ability of the research findings. The psychological development and teaching management models of students at different stages, such as university, high school, and junior high school, vary considerably. Due to limitations in research duration, manpower, and funding, this study only selected university students as the research subjects, resulting in a less comprehensive sample representation and limitations in the conclusions drawn. Future research could expand the sample scope to generalize the research findings to a wider population. (4) This study examined the mediating role of ego depletion in the relationship between state anxiety and academic procrastination among college students, as well as the moderating role of student gender. In the next step, potential interactions between gender, ego depletion, and other variables could be considered for discussion, in order to enrich new discoveries and impact mechanisms regarding academic procrastination. Additionally, trait anxiety and state anxiety, though seemingly different, are closely intertwined. Whether they jointly contribute to academic procrastination behavior is a question that the research team intends to further explore in the next phase of the study.

## Conclusions

The conclusions of this study are as follows: (1) State anxiety significantly and positively predicts academic procrastination among university students. (2) Ego depletion partially mediates the relationship between state anxiety and academic procrastination. (3) The direct predictive function of state anxiety on academic procrastination and the mediating role of ego depletion are both moderated by gender. State anxiety exerts a stronger influence on academic procrastination in male individuals, while ego depletion has a more pronounced impact on academic procrastination in the female population.

For future research, the following studies can be carried out on the basis of this finding: (1) Utilize specific longitudinal or experimental design methods to further explore the extent of academic procrastination among college students under different anxiety levels, delve into the mechanism of how state anxiety affects academic procrastination behaviors, and thereby establish a social support system for students with academic procrastination issues, aiming to better address the problem of academic procrastination among college students. (2) As there are differences in psychological development and teaching management models among college students, high school students, junior high school students, and other student groups, future research needs to expand the sample range to generalize the research results to a wider student population. (3) Trait anxiety and state anxiety seem different but are closely related. Whether they jointly influence academic procrastination behaviors is an important issue that needs to be explored in the next step.

## Data Availability

The datasets generated and analysed during the current study are not publicly available due to limitations of university policy and anonymity of the questionnaire we conducted, but are available from the corresponding author on reasonable request.
